# Prevalence of Musculoskeletal Disorders and Associated Risk Factors Among Professional Drivers in Chromepet, Chengalpattu District: A Cross-Sectional Study

**DOI:** 10.7759/cureus.85655

**Published:** 2025-06-09

**Authors:** Gokul Gopakumar, Aljin V, Hariharan Surathkumaar, Ramkumar T, Subhashini Viswanath, Jeffrey Joseph

**Affiliations:** 1 Community Medicine, Sree Balaji Medical College and Hospital, Chennai, IND; 2 Community Medicine, Meenakshi Medical College Hospital and Research Institute, Meenakshi Academy of Higher Education and Research (Deemed to be University), Kanchipuram, IND; 3 Community Medicine, Sree Balaji Medical College and Hospital, Bharath Institute of Higher Education and Research, Chennai, IND

**Keywords:** chronic pain, human factors and ergonomics, increased work load, musculoskeletal abnormality, professional drivers

## Abstract

Background

Professional drivers frequently suffer from musculoskeletal disorders (MSDs) due to prolonged sitting, poor posture, repetitive movements, and exposure to whole-body vibrations. These factors contribute to pain and discomfort, commonly affecting the shoulders, limbs, back, and neck. Professional drivers are particularly vulnerable because of extended driving hours, exposure to vibration, and awkward postures. The objective of this study is to assess the prevalence and patterns of musculoskeletal morbidity among professional drivers and to identify the factors influencing its occurrence.

Methods

A cross-sectional study was conducted among 264 professional drivers in the Chromepet area. Participants were recruited through travel agencies, and data were collected using an interviewer-administered questionnaire that included sociodemographic details, personal habits, work characteristics, and the Nordic Musculoskeletal Questionnaire to assess pain distribution. Descriptive data were analyzed using frequencies and percentages, and logistic regression was used to identify factors associated with MSDs.

Results

The study population consisted of 249 (94.32%) males and 15 (5.68%) females. Chronic pain was most commonly reported in the upper back (213; 80.7%), lower back (210; 79.5%), shoulders (208; 78.8%), and neck (203; 76.9%). Prolonged daily shift hours significantly increased the risk of musculoskeletal pain, with those working 12-16 hours having the highest odds for neck pain (OR = 11.73, p = 0.006), upper back pain (OR = 23.93, p = 0.001), and lower back pain (OR = 8.53, p = 0.010). Longer job duration was also associated with increased risk of upper and lower back pain.

Conclusion

The prevalence of MSDs among professional drivers in Chromepet is high, particularly in the back and shoulder regions. Longer job duration and extended working hours significantly increase the risk of developing MSDs. These findings highlight the need for targeted ergonomic interventions and health promotion strategies to reduce the burden of MSDs among professional drivers.

## Introduction

Musculoskeletal disorders (MSDs) encompass a wide range of conditions affecting the muscles, bones, joints, tendons, ligaments [1], cartilage, rheumatoid arthritis [2], osteoarthritis, neck pain, and spinal disks [3-6]. These disorders can result in pain, discomfort, and significant limitations in mobility and functionality. Globally, approximately 1.71 billion people live with MSDs, making them a leading cause of disability and a significant public health concern [7,8]. The Global Burden of Disease Study 2019 highlighted that MSDs ranked fifth among all diseases in terms of disability-adjusted life years (DALYs), indicating their substantial impact on quality of life [8].

The prevalence of MSDs varies by region, with high-income countries reporting the highest numbers. For instance, 441 million individuals in high-income countries are affected, followed by 427 million in the Western Pacific region and 369 million in South-East Asia [7]. Low back pain is the most prevalent condition within this category, contributing significantly to the global burden of disease [8,9].

Professional drivers are particularly vulnerable to developing MSDs due to several occupational factors. These include prolonged periods of sitting, exposure to whole-body vibrations, and the necessity of maintaining awkward postures while driving. Research indicates that the prevalence of musculoskeletal pain (MSP) among professional drivers can range from 43.1% to 93%, with low back pain being the most frequently reported issue, affecting over 53% of drivers in various studies [10-12].

Several studies have explored MSD prevalence among professional drivers in different contexts. A systematic review indicated that bus drivers have a prevalence rate of 80%, while truck drivers report rates around 81% [12,13]. Research conducted in metropolitan areas highlighted significant disparities in MSD rates between professional drivers and office workers, emphasizing the need for tailored health strategies [13-15]. A meta-analysis confirmed that low back pain is prevalent among professional drivers across various studies, reinforcing the need for targeted preventive measures [16,17]. This study aims to fill the gap in literature by providing specific data on MSD prevalence among professional drivers in Chromepet, Chengalpattu district. The main objectives of the study were to assess the prevalence and pattern of musculoskeletal morbidity among professional drivers, and to determine the factors associated with the occurrence of musculoskeletal morbidity in the study group. By focusing on this under-researched population, we hope to contribute valuable insights that can inform local health policies and interventions aimed at reducing the incidence of MSDs among professional drivers.

## Materials and methods

This cross-sectional study was conducted among professional drivers in the Chromepet area of Chengalpattu district.

The study population included both male and female professional drivers with at least one year of driving experience who were willing to participate. Participants were recruited through travel agencies; there are 50 travel agencies in and around the Chromepet area. Among them, 26 agencies that gave permission to conduct the study were selected by simple random sampling. The line list of drivers operating cars, buses, and delivery trucks was obtained from the records of these agencies. Participants who gave consent for the study were included. After obtaining permission from the agencies, the purpose of the study was explained to participants, and those who gave consent were included in the study.

Data collection was carried out by directly approaching the drivers after obtaining their signed informed consent. An interviewer-administered, semi-structured questionnaire was used for data collection on sociodemographic characteristics, as well as participants’ personal and medical history. MSD pain distribution was captured using the Nordic scale [18]. The study was conducted over a period of six months, from July 2024 to December 2024. In this study, individuals having any of the following conditions, diabetes, hypertension, and cancer, for more than six months were classified under “chronic conditions.”

Descriptive data were analyzed using frequencies and percentages. Factors affecting each pain area were analyzed using logistic regression. Ethical committee approval was obtained from the Institutional Human Ethics Committee of Sree Balaji Medical College and Hospital, with reference number 002/SBMCH/IHEC/2023/2100.

As per a study on work-related MSDs, the prevalence was found to be 33.8% in a study by Reddy GM et al. [19]. The sample size was calculated using the formula below, with an absolute error of 6%:

P= 33.8% q= 66.2 z=1.96 L=6 N= z²pq / L² 

N = 1.96x1.96x33.8x66.2/6x6 = 238.77, sample size rounded to 264.

The data were entered in an Excel sheet, and data analysis was performed using SPSS software version 22.

## Results

Table 1 shows that out of 264 participants, the majority were male (249; 94.32%), with females accounting for 15 (5.68%). In terms of marital status, 147 (55.7%) were married, followed by 36 (13.6%) separated/divorced, 75 (28.4%) single, and 6 (2.3%) widowed. Regarding monthly household income, the largest group (122; 46.2%) reported earning ₹9097 and above, while 87 (33%) earned between ₹4549-9097, and 45 (17%) earned between ₹2729-4550. Fewer participants were in the lower income brackets, with 2 (0.8%) earning below ₹1365 and 8 (3%) earning between ₹1365-2728. In terms of education, 8 (3%) participants were illiterate. Among those with formal education, 47 (17.8%) had completed high school, 52 (19.7%) middle school, 75 (28.4%) intermediate or diploma-level education, 20 (7.6%) primary school, and 47 (17.8%) were graduates. A smaller proportion (15; 5.7%) had professional degrees.

**Table 1:** Sociodemographic characteristics of study participants (n = 264).

Variable	Categories	Frequency (N = 264)	Percentage (%)
Gender	Male	249	94.32
	Female	15	5.68
Marital Status	Married	147	55.7
	Separated/Divorced	36	13.6
	Single	75	28.4
	Widowed	6	2.3
Monthly Household Income	Below ₹1,365	2	0.8
	₹1,365-2,728	8	3
	₹2,729-4,550	45	17
	₹4,549-9,097	87	33
	₹9,097 and above	122	46.2
Educational Qualification	Illiterate	8	3
	Primary School	20	7.6
	Middle School	52	19.7
	High School	47	17.8
	Intermediate/Diploma	75	28.4
	Graduate	47	17.8
	Professional Degree	15	5.7
Type of Family	Nuclear	143	54.2
	Joint	47	17.8
	Three-Generation Family	74	28
Health Insurance Taken	Yes	97	36.7
	No	167	63.3

Table 2 shows that smoking was reported by 57 (21.6%) participants, while the remaining 207 (78.4%) were non-smokers. Similarly, 55 (20.8%) reported consuming alcohol, while 209 (79.2%) did not. Physical activity levels were low, with only 70 (26.5%) engaging in regular physical activity, while 194 (73.5%) reported no such activity.

**Table 2:** Personal habits of study participants (n = 264).

Variable	Categories	Frequency	Percentage
Smoking	Yes	57	21.60%
	No	207	78.40%
Alcohol	Yes	55	20.80%
	No	209	79.20%
Physical Activity	Yes	70	26.50%
	No	194	73.50%

Table 3 shows that the largest proportion of participants (115; 43.6%) worked 8-12 hours per day, followed by 74 (28.0%) working 12-16 hours. Regarding breaks, most participants (162; 61.4%) had 2-4 hours of break time during work, while 93 (35.2%) had 1-2 hours. Around 83 (31.4%) participants reported being exposed to vibration during work, while the remaining 181 (68.6%) were not. Regarding the duration of employment in their current job, 83 (31.4%) had been employed for 2-4 years, while 80 (30.3%) reported more than six years. When considering commuting distance, the majority (153; 58%) worked within short distances, while 56 (21.2%) traveled both short and long distances, and 55 (20.8%) traveled long distances exclusively.

**Table 3:** Work-related characteristics of study participants (n = 264).

Variable	Categories	Frequency	Percentage
Daily shift hours	1-4 hrs	7	2.7
	4-8 hrs	42	15.9
	8-12 hrs	115	43.6
	12-16 hrs	74	28.0
	16-24 hrs	26	9.8
Break hours during work	1-2 hrs	93	35.2
	2-4 hrs	162	61.4
	4-8 hrs	9	3.4
Vibration of the vehicle	Yes	83	31.4
	No	181	68.6
Duration of present job	>6 years	80	30.3
	4-6 yrs	36	13.6
	2-4 yrs	83	31.4
	1-2 yrs	65	24.7
Distance	Short distance (<20 km)	153	58.0
	Short and long distance (20-40 km)	56	21.2
	Long distance (>40 km)	55	20.8

Table 4 shows that among the participants, 30 (11.4%) reported having chronic illnesses, while 234 (88.6%) reported no chronic illnesses. Over-the-counter medications were consumed by 40 (15.2%) participants, and 190 (72%) preferred government hospital facilities for their healthcare needs.

**Table 4:** Health-seeking behavior among study participants (n = 264).

Variable	Categories	Frequency	Percentage
Any chronic condition	Yes	30	11.4
	No	234	88.6
Any intake of painkillers over the counter	Yes	40	15.2
	No	224	84.8
Choice of healthcare facility for illness	Government hospital	190	72.0
	Private hospital	74	28.0

Table 5 shows that the most common chronic pain was reported in the neck (203; 76.9%), upper back (213; 80.7%), lower back (210; 79.5%), and shoulder (208; 78.8%) regions.

**Table 5:** One-year regional pain distribution among study participants (n = 264).

Region	Male (%)	Female (%)	Total (%)
Neck	189 (93.1)	14 (6.9)	203 (76.9)
Shoulder	195 (93.8)	13 (6.3)	208 (78.8)
Upper back	200 (93.9)	13 (6.1)	213 (80.7)
Elbow	130 (94.9)	7 (5.1)	137 (51.9)
Wrist/Hand	128 (92.8)	10 (7.2)	138 (52.3)
Lower back	196 (93.3)	14 (6.7)	210 (79.5)
Hips/Thighs	113 (94.2)	7 (5.8)	120 (45.5)
Knees	132 (94.3)	8 (5.7)	140 (53.0)
Ankles/Feet	146 (93.6)	10 (6.4)	156 (59.1)

Table 6 shows the binary logistic regression analysis revealing associations between occupational factors and musculoskeletal pain.

**Table 6:** Factors associated with musculoskeletal disorders in the most commonly affected body regions over the past 12 months: Binary logistic regression with unadjusted odds ratios among study participants (n = 264). *Statistically significant at 95% CI; Binary logistic regression analysis was used.

Pain area	Variable	Categories	OR	95% CI Lower	95% CI Upper	P-value
Neck	Smoking	Yes	0.635	0.329	1.226	0.174
	Physical activity	Yes	0.670	0.359	1.249	0.206
	Any chronic disease	Yes	0.287	0.131	0.630	0.001^*^
	Vibration of the vehicle	Yes	1.018	0.549	1.887	0.955
	Daily shift hours	1-4 hrs	Reference category			
		4-8 hrs	5.577	0.954	32.594	0.056
		8-12 hrs	8.558	1.568	46.706	0.013*
		12-16 hrs	11.731	2.047	67.217	0.006*
		16-24 hrs	13.750	1.946	97.178	0.009*
	Duration of present job	>6 yrs	0.724	0.336	1.561	0.410
		4-6yrs	0.961	0.359	2.569	0.936
		2-4yrs	1.066	0.481	2.363	0.875
		1-2yrs	Reference category			
Upper back	Smoking	Yes	0.291	0.150	0.564	0.000*
	Physical activity	Yes	0.592	0.308	1.139	0.114
	Any chronic disease	Yes	0.254	0.114	0.565	0.000*
	Vibration of the vehicle	Yes	1.004	0.520	1.939	0.991
	Daily shift hours	1-4 hrs	Reference category			
		4-8 hrs	4.063	0.703	23.474	0.117
		8-12 hrs	14.412	2.584	80.384	0.002*
		12-16 hrs	23.929	3.896	146.984	0.001*
		16-24 hrs	8.333	1.267	54.423	0.027*
	Duration of present job	>6 yrs	0.917	0.432	1.947	0.822
		4-6yrs	5.551	1.198	25.730	0.028*
		2-4yrs	1.932	0.841	4.441	0.121
		1-2yrs	Reference category			
Lower back	Smoking	Yes	0.456	0.235	0.886	0.019*
	Physical activity	Yes	0.478	0.254	0.901	0.021*
	Any chronic disease	Yes	0.463	0.203	1.059	0.063
	Vibration of the vehicle	Yes	0.897	0.474	1.695	0.737
	Daily shift hours	1-4 hrs	Reference category			
		4-8 hrs	2.974	0.581	15.236	0.191
		8-12 hrs	5.636	1.176	27.022	0.031*
		12-16 hrs	8.533	1.658	43.929	0.010*
		16-24 hrs	5.600	0.938	33.428	0.059
	Duration of present job	>6 yrs	1.333	0.642	2.768	0.440
		4-6yrs	3.556	1.109	11.402	0.033*
		2-4yrs	3.244	1.394	7.554	0.006*
		1-2yrs	Reference category			
Shoulder pain	Smoking	Yes	0.389	0.203	0.748	0.004*
	Physical activity	Yes	0.570	0.303	1.072	0.079
	Any chronic disease	Yes	0.489	0.215	1.116	0.085
	Vibration of the vehicle	Yes	1.066	0.562	2.022	0.844
	Daily shift hours	1-4 hrs	Reference category			
		4-8 hrs	2.266	0.382	13.434	0.368
		8-12 hrs	9.192	1.616	52.275	0.012*
		12-16 hrs	14.899	2.353	94.343	0.004*
		16-24 hrs	9.273	1.304	65.956	0.026*
	Duration of present job	>6 yrs	0.966	0.423	2.206	0.935
		4-6yrs	2.040	0.595	6.986	0.257
		2-4yrs	2.085	0.874	4.972	0.097
		1-2yrs	Reference category			

Neck pain

Prolonged daily shift hours were a strong predictor of neck pain. Compared to those working 1-4 hours, individuals working 8-12 hours (OR = 8.56, p = 0.013), 12-16 hours (OR = 11.73, p = 0.006), and 16-24 hours (OR = 13.75, p = 0.009) had significantly higher odds of experiencing neck pain.

Upper back pain

Similar trends were observed for upper back pain, with significantly increased odds for those working 8-12 hours (OR = 14.41, p = 0.002), 12-16 hours (OR = 23.93, p = 0.001), and 16-24 hours (OR = 8.33, p = 0.027). Additionally, workers with a job duration of 4-6 years were at a higher risk of upper back pain (OR = 5.55, p = 0.028).

Lower back

Lower back pain was also associated with longer working hours (8-12 hours: OR = 5.64, p = 0.031; 12-16 hours: OR = 8.53, p = 0.010) and longer job duration (4-6 years: OR = 3.56, p = 0.033; 2-4 years: OR = 3.24, p = 0.006).

Shoulder pain

Compared to those working 1-4 hours per day, individuals working longer shifts had higher odds of shoulder pain. The risk was particularly significant for 8-12 hours (OR = 9.192, p = 0.012), 12-16 hours (OR = 14.899, p = 0.004), and 16-24 hours (OR = 9.273, p = 0.026), indicating a strong association between prolonged work hours and shoulder pain. However, no statistically significant association was observed between job duration and shoulder pain. The odds ratios for different durations (e.g., 2-4 years: OR = 2.085, p = 0.097; 4-6 years: OR = 2.040, p = 0.257) suggest a potential trend, but the findings are not conclusive.

## Discussion

The findings regarding MSDs among professional drivers in Chromepet, Chengalpattu district, reveal significant insights into the prevalence and risk factors associated with chronic pain in various body regions. The reported high prevalence of pain in the neck 203 (76.9%), upper back 213 (80.7%), lower back 210 (79.5%), and shoulder 208 (78.8%) aligns with existing literature that emphasizes the occupational hazards faced by drivers due to prolonged sitting, poor ergonomics, and lack of physical activity.

The prevalence rates of MSDs in this study are consistent with previous research indicating that professional drivers are at a heightened risk for developing neck and back pain. For instance, a study conducted on bus drivers found that 26% reported neck pain and 24% reported back pain, highlighting similar trends in occupational health issues among drivers [20-21]. The high prevalence of upper back pain among drivers can be attributed to prolonged exposure to static postures and vibrations from the vehicle, which have been shown to contribute significantly to musculoskeletal discomfort [22].

The study found that drivers working longer shifts, specifically 8-12 hours, 12-16 hours, and 16-24 hours, exhibited significantly higher odds of experiencing neck and upper back pain. Neck pain increased dramatically with longer shifts, indicating a clear relationship between extended work hours and discomfort. A systematic review reported that professional drivers have a high prevalence of MSDs due to risk factors such as prolonged sitting, whole-body vibration, and awkward postures, with low back pain being the most frequently reported condition (meta-prevalence rate of 53%) among drivers globally [23].

The association between job duration and upper back pain is particularly noteworthy for those with 4-6 years of experience. This suggests that longer exposure to occupational hazards may increase the risk of developing MSDs. Research has shown that cumulative exposure to physical stressors can lead to degenerative changes in the musculoskeletal system. For example, a study among taxi drivers indicated an overall prevalence of MSDs at 86.8%, highlighting the impact of job tenure on pain prevalence [24-26]. Furthermore, truck drivers with extensive experience reported higher rates of low back pain due to prolonged exposure to vibrations and sustained postures [16].

Lower back pain was significantly associated with longer working hours and a job duration of 4-6 years. These findings align with literature suggesting that prolonged sitting and repetitive movements are major contributors to lower back discomfort among drivers [27-28].

Interestingly, the study found that smoking was associated with a lower risk of pain at all major sites. This could be due to the action of nicotine on the CNS, which may modulate pain perception [29]. The presence of chronic disease was also linked to reduced odds of neck and upper back pain. However, this does not imply a protective effect of these factors, and further research is needed to explore potential confounding variables influencing this association.

The implications of these findings are significant for occupational health interventions targeting professional drivers. Ergonomic assessments and modifications in driver seating arrangements could help mitigate risks associated with prolonged sitting and exposure to vibrations. Additionally, promoting physical activity through structured exercise programs could serve as an effective preventive strategy against MSDs.

Strength and limitations

This study provides valuable insights into the prevalence and risk factors of MSDs among professional drivers in Chromepet, Chengalpattu district. One of its main strengths lies in its focus on a high-risk occupational group, the use of standardized data collection tools, and an adequate sample size that enhances the reliability of findings. However, the cross-sectional design limits causal inference. Self-reported data may introduce recall bias, and the results may not be generalizable beyond the study area. Furthermore, unmeasured factors such as lifestyle choices and psychological stress may have influenced the outcomes. Despite these limitations, the study offers important evidence to inform occupational wellness interventions.

## Conclusions

The study reveals a high prevalence of MSDs among professional drivers in Chromepet, Chengalpattu district, with the neck, upper back, lower back, and shoulders being the most commonly affected regions. Factors such as job duration, smoking habits, and lack of physical activity were found to significantly influence the occurrence of musculoskeletal pain, particularly in the back and neck areas. These findings highlight the need for targeted occupational health interventions, including ergonomic improvements in driving posture, promotion of regular physical activity, and smoking cessation programs, to mitigate the risk of MSDs among professional drivers. This study provides valuable insights into the specific health challenges faced by this vulnerable population and underscores the importance of tailored preventive strategies to improve their musculoskeletal health and overall well-being.

## Appendices

1. Name

2. Age

3. Gender Male Female

4. Address

5. Marital status Single Married Divorced Seperated Widowed

6. Montly household income Rs 9098 & above Rs 4549 - 9097 Rs 2729 - 4550 Rs 1365 - 2728 below Rs 1365

7. Educational qualification Professional degree Graduate Intermediate/Diploma High school Middle school Primary school Illiterate

8. Socioeconomic status

9. Type of family Nuclear Joint family Three generation family

10. Number of family members

11. Any Health Insurance Yes No

12. Smoking Yes No

13. Alcohol Yes No

14. Physical Activity Yes No

15. Employment (years)

16. Any Chronic diseases Yes No

17. Type of vehicle Bike Car Auto rikshaw Pickup van Tempo traveller Bus Lorry

18. Daily Shift (hours) 1 - 4 hours 4 - 8 hours 8 - 12 hours 12 - 16 hours 16 - 24 hours

19. How many breaks (hours) 1 - 2 hours 2 - 4 hours 4 - 8 hours

20. Vibration Yes No

21. Duration of Present Job 6 months - 1 year 1 year - 2 years 2 years - 4 years 4 years - 6 years > 6 years

22. Distance Short Distance Long Distance Short and long distance

23. Any Intake of painkillers over the counter Yes No

24. Any postural problems Yes No

25. Any Visual Problems Pain in eyes Watering from eyes Headache Nil

26. Any Genito Urinary problems Recurrent UTI Sexually transmitted Infections Nil

27. Any Respiratory Problems Coughing/Sneezing Dysnea/Asthma Nil

28. Any Gastroeneterology Problems Recurrent Diarrhea Constipation Acid peptic Disease Nil

29. Any Dermatology Condition Skin allergy/Dryness Scabies Diseases of Hair/Nails Nil

30. Where do you prefer to visit for Illness Government Hospital Private Hospital

31. Others Stress Tension Fatigue Insomnia Job stress Job Support Job dissatisfaction Nil

Nordic Scale

**Neck** 32. Have you at any time during the last 12 months had trouble (such as ache,pain, discomfort,numbness) NO YES

33. During the last 12 months have you been prevented from carrying out normal activities (e.g. job, housework, hobbies) because of this trouble in No Yes

34. During the last 12 months have you seen a physician for this condition No Yes

35. During the last 7 days have you had trouble in: No Yes

**Shoulder** 36. Have you at any time during the last 12 months had trouble (such as ache,pain, discomfort,numbness) NO YES

37. During the last 12 months have you been prevented from carrying out normal activities (e.g. job, housework, hobbies) because of this trouble in No Yes

38. During the last 12 months have you seen a physician for this condition No Yes

39. During the last 7 days have you had trouble in: No Yes

**Upper Back** 40. Have you at any time during the last 12 months had trouble (such as ache,pain,discomfort,numbness) NO YES

41. During the last 12 months have you been prevented from carrying out normal activities (e.g. job, housework, hobbies) because of this trouble in No Yes

42. During the last 12 months have you seen a physician for this condition No Yes

43. During the last 7 days have you had trouble in No Yes

**Elbow** 44. Have you at any time during the last 12 months had trouble (such as ache,pain, discomfort,numbness) NO YES

45. During the last 12 months have you been prevented from carrying out normal activities (e.g. job, housework, hobbies) because of this trouble in No Yes

46. During the last 12 months have you seen a physician for this condition No Yes

47. During the last 7 days have you had trouble in No Yes

**Wrist/Hands** 48. Have you at any time during the last 12 months had trouble (such as ache,pain, discomfort,numbness) NO YES

49. During the last 12 months have you been prevented from carrying out normal activities (e.g. job, housework, hobbies) because of this trouble in No Yes

50. During the last 12 months have you seen a physician for this condition No Yes

51. During the last 7 days have you had trouble in No Yes

**Lowerback** 52. Have you at any time during the last 12 months had trouble (such as ache,pain, discomfort,numbness) NO YES

53. During the last 12 months have you been prevented from carrying out normal activities (e.g. job, housework, hobbies) because of this trouble in No Yes

54. During the last 12 months have you seen a physician for this condition No Yes

55. During the last 7 days have you had trouble in No Yes

**Hips/Thighs** 56. Have you at any time during the last 12 months had trouble (such as ache,pain, discomfort,numbness) NO YES

57. During the last 12 months have you been prevented from carrying out normal activities (e.g. job, housework, hobbies) because of this trouble in No Yes

58. During the last 12 months have you seen a physician for this condition No Yes

59. During the last 7 days have you had trouble in No Yes

**Knees** 60. Have you at any time during the last 12 months had trouble (such as ache,pain, discomfort,numbness) NO YES

61. During the last 12 months have you been prevented from carrying out normal activities (e.g. job, housework, hobbies) because of this trouble in No Yes

62. During the last 12 months have you seen a physician for this condition No Yes

63. During the last 7 days have you had trouble in No Yes

**Ankles/Feet** 64. Have you at any time during the last 12 months had trouble (such as ache,pain, discomfort,numbness) NO YES

65. During the last 12 months have you been prevented from carrying out normal activities (e.g. job, housework, hobbies) because of this trouble in No Yes

66. During the last 12 months have you seen a physician for this condition No Yes

67. During the last 7 days have you had trouble in No Yes
